# Assessment of Variability in Cerebral Blood Flow and Cerebral Blood Volume in Cerebral Arteries of Ischemic Stroke Patients Using Dynamic Contrast-Enhanced MRI

**DOI:** 10.3390/tomography11110117

**Published:** 2025-10-22

**Authors:** Bilal Bashir, Babar Ali, Saeed Alqahtani, Benjamin Klugah-Brown

**Affiliations:** 1 Single Molecule Imaging Lab, Institute of Fundamental and Frontier Sciences (IFFS), University of Electronic Science and Technology of China (UESTC), Chengdu 610054, China; bilalbashir8757@gmail.com; 2 University Institute of Radiological Sciences and Medical Imaging Technology, The University of Lahore, Lahore 54000, Pakistan; babarali0741@gmail.com; 3 Radiological Sciences Department, College of Applied Medical Sciences, Najran University, Najran 61441, Saudi Arabia; 4 MOE Key Laboratory for Neuroinformation, School of Life Science and Technology (SLST), University of Electronic Science and Technology of China (UESTC), Chengdu 610054, China; bklugah@uestc.edu.cn

**Keywords:** cerebral blood flow (CBF), cerebral blood volume (CBV), ischemic stroke, dynamic contrast-enhanced MRI (DCE-MRI), cerebral arteries, age, gender

## Abstract

Background/Objectives: Cerebral blood flow (CBF) and cerebral blood volume (CBV) are critical perfusion metrics in diagnosing ischemic stroke. Dynamic contrast-enhanced magnetic resonance imaging (DCE-MRI) enables the evaluation of these cerebral perfusion metrics; however, accurately assessing them remains challenging. This study aimed to: (1) assess CBF asymmetry by quantifying and comparing it between contralateral hemispheres (right vs. left) within the MCA, ACA, and PCA territories using paired *t*-tests, and describe pattern of CBV; (2) evaluate overall inter-territorial regional variations in CBF across the different cerebral arterial territories (MCA, ACA, PCA), irrespective of the hemisphere, using ANOVA; (3) determine the correlation between CBF and CBV using both Pearson’s and Spearman’s correlation analyses; and (4) assess the influence of age and gender on CBF using multiple regression analysis. Methods: A cross-sectional study of 55 ischemic stroke patients was conducted. DCE-MRI was used to measure CBF and CBV. Paired *t*-tests compared contralateral hemispheric CBF in MCA, PCA, and ACA, one-way ANOVA assessed overall inter-territorial CBF variations, correlation analyses (Pearson/Spearman) evaluated the CBF-CBV relationship, and linear regression modeled demographic effects. Results: Significant contralateral asymmetries in CBF were observed for each cerebral pair of cerebral arteries using a paired *t*-test, with descriptive asymmetries noted in CBV. Separately, ANOVA revealed significant overall variability in CBF between the different cerebral arteries, irrespective of hemisphere. A strong positive correlation was found between CBF and CBV (Pearson r = 0.976; Spearman r = 0.928), with multiple regression analysis identifying age and gender as significant predictors of CBF. Conclusions: This study highlights hemispheric asymmetry and inter-territorial variation, the impact of age, and gender on CBF. DCE-MRI provides perfusion metrics that can guide individualized stroke treatment, offering valuable insights for therapeutic planning, particularly in resource-limited settings.

## 1. Introduction

Ischemic stroke is a serious and time-sensitive neurological condition that results from the obstruction of blood supply to brain tissue, accounting for approximately 85% of all stroke cases globally [1,2,3]. It leads to a cascade of metabolic failure and neuronal injury due to reduced cerebral perfusion and oxygen deprivation [4,5]. Stroke-related morbidity and mortality are especially concerning in low- and middle-income countries (LMICs), where resource limitations delay diagnosis and limit access to advanced neuroimaging [2,6].

Cerebral perfusion parameters, CBF and CBV, are critical indicators of tissue viability in ischemic stroke and are used to distinguish reversible ischemia from irreversible infarction [7,8,9]. Accurate measurement of these parameters aids in identifying viable penumbral tissue (the area of the brain at risk but not yet infarcted) and tailoring timely therapeutic interventions [3,10]. While CT perfusion (CTP) and dynamic susceptibility contrast MRI (DSC-MRI) remain the most common modalities used for perfusion imaging, they have limitations in terms of radiation exposure and reliance on susceptibility effects [11,12].

An emerging alternative is DCE-MRI, which leverages T1-weighted imaging and time-resolved contrast kinetics to generate semi-quantitative estimations of perfusion metrics [13,14,15]. DCE-MRI offers an enhanced signal-to-noise ratio, does not rely on susceptibility artefacts, and allows post-processing of pharmacokinetic data, making it a promising tool in stroke research and management [16,17]. Several studies have highlighted the potential of DCE-MRI to evaluate perfusion dynamics across major vascular territories of the brain in stroke patients [18,19,20].

However, a review of existing literature reveals critical research gaps: (1) most perfusion studies are focused on DSC-MRI or CT perfusion techniques [11,12,21]; (2) few investigations have employed DCE-MRI to evaluate CBF and CBV in ischemic stroke, particularly in low and middle-income countries (LMICs) [13,16,22]; and (3) the effects of regional asymmetry (left vs. right hemisphere) and demographic variables such as age and gender on CBF and CBV remain underexplored using DCE-MRI [13,18,23].

While several prior studies have reported that CBF and CBV vary across vascular territories, with differences between right and left hemispheres of MCA, ACA, and PCA [15,24,25], these findings have relied mainly on CT or DSC-MRI modalities. Although a limited number of studies have begun to explore age- and sex-related perfusion differences using DCE-MRI [3,19,20]. Consequently, a significant gap persists in studies using DCE-MRI to investigate perfusion asymmetries and demographic influences, especially in low- and middle-income countries (LMICs), such as Pakistan. This study addresses this gap by providing regional and demographic perfusion analysis in a Pakistani ischemic stroke (AIS) cohort using DCE-MRI.

This study addresses the gaps identified in the existing literature by using DCE-MRI to evaluate cerebral perfusion in a cohort of ischemic stroke patients from Pakistan. Specifically, the objectives were: (1) To identify significant CBF asymmetries by quantifying and comparing it between contralateral hemispheres (right vs. left) within the same arterial territory (i.e., right MCA vs. left MCA) using paired *t*-tests, and CBV descriptively to identify significant perfusion asymmetries; (2) to evaluate overall inter-territorial variations in CBF across the different cerebral arterial territories (MCA, ACA, PCA), irrespective of the hemisphere, using one-way analysis of variance (ANOVA); (3) to determine the correlation between CBF and CBV using both Pearson’s and Spearman’s correlation analyses; and (4) to assess the influence of demographic variables (age and gender) on CBF using multiple regression analysis.

Based on the prior literature we hypothesized that: (H1) significant differences in CBF and CBV would be observed between contralateral cerebral arteries (MCA, ACA, PCA), reflecting potential hemispheric lateralization in ischemic stroke perfusion patterns; (H2) CBF would vary significantly across the MCA, ACA, and PCA territories, for example, due to inherent vascular heterogeneity; (H3) a strong positive correlation would exist between CBF and CBV; and (H4) increasing age and male gender would be significant predictors of lower CBF.

To the best of our knowledge, this study represents the first DCE-MRI-based evaluation of cerebral perfusion in the ischemic stroke patient population from Pakistan. It provides baseline demographic-specific data on CBF and CBV, which may enhance stroke imaging accuracy, inform clinical decision-making in resource-limited settings, and support the development of personalized therapeutic strategies.

## 2. Materials and Methods

### 2.1. Study Design and DCE-MRI Protocol

This cross-sectional, single-center observational study was conducted at the Department of Radiology, The University of Lahore Teaching Hospital, Lahore, Punjab, Pakistan, from July 2024 to January 2025. The primary objective was to assess cerebral perfusion dynamics, including CBF and CBV, in ischemic stroke, using a dynamic contrast-enhanced DCE-MRI protocol.

All imaging was performed within 48 h of stroke onset, capturing the hyperacute and acute phases of ischemia. Patients were diagnosed through clinical neurological examination and documentation provided by the referring physician. The DCE-MRI protocol, using time-resolved T1-weighted sequences and intravenous contrast, was employed to evaluate regional perfusion in anatomically defined arterial territories of the brain, including the MCA, ACA, and PCA.

Although DCE-MRI is more commonly used in clinical contexts such as tumor imaging or vascular assessments, it was chosen for this study due to its institutional accessibility and its ability to provide time-resolved perfusion data. The values of CBF and CBV were derived using a semi-quantitative approach based on basic pharmacokinetic principles of contrast dynamics. The study also explored the influence of age and gender on CBF variations in ischemic stroke patients.

Although DCE-MRI is more commonly used in clinical contexts such as tumor imaging or vascular assessments, it was chosen for this study due to its institutional accessibility and its ability to generate temporal enhancement curves, from which semi-quantitative estimates of CBF and CBV were derived. These values were extracted through contrast enhancement dynamics and basic pharmacokinetic modelling applied to defined regions of interest (ROIs). The study also explored the influence of demographic factors, such as age and gender, on CBF variations in ischemic stroke patients.

### 2.2. Study Cohort and Inclusion/Exclusion Criteria

**Figure 1:**
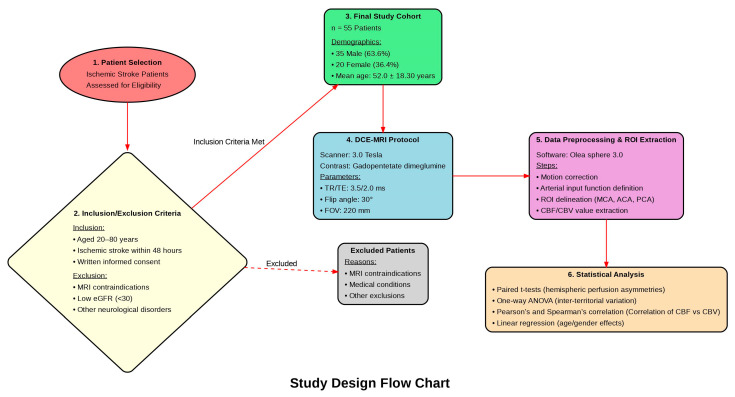
Study design flow chart for the assessment of cerebral perfusion in ischemic stroke patients using Dynamic Contrast-Enhanced MRI. The diagram outlines the sequential process from initial patient screening through final statistical analysis. It begins with eligibility assessment of ischemic stroke patients, application of inclusion/exclusion criteria, formation of the final cohort (n = 55), DCE-MRI acquisition protocol specification, data preprocessing with region of interest (ROI) extraction in major cerebral arteries (Middle Cerebral Artery, Anterior Cerebral Artery, Posterior Cerebral Artery), and concludes with the statistical analysis plan for evaluating perfusion asymmetries, inter-territorial variations, and demographic effects.

A convenience sample of ischemic stroke patients was recruited from the Department of Neuroradiology during the study period. Eligible participants were aged between 20 and 80 years, had experienced a clinically diagnosed ischemic stroke within 48 h of symptom onset, and provided written informed consent before participation. Stroke diagnosis was established based on clinical presentation and referral documentation from the attending neurologist. As shown in Figure 1

The age range for inclusion was set between 20 and 80 years to focus on adults with ischemic stroke while excluding the potential confounding effects of age-related comorbidities. Although older patients are often included in stroke studies, individuals over the age of 80 were excluded from this study to minimize the potential impact of age-related comorbidities, such as severe cardiovascular diseases, cognitive decline, and other systemic conditions, which are more prevalent in this age group and could confound the interpretation of cerebral perfusion dynamics. The sample size of 55 participants was selected based on the resources available at the study site, the institutional capacity for patient recruitment, and the practical considerations of conducting the research within the given timeline. Although power analysis was not performed, the sample size was determined to be adequate for the study’s objectives, based on clinical guidelines, prior studies, and resource constraints.

Exclusion criteria included the presence of any contraindication to MRI, such as metallic implants, severe claustrophobia, or inability to receive gadolinium-based contrast agents (GBCAs). Participants with an estimated glomerular filtration rate (eGFR) below 30 mL/min/1.73 m^2^, known contrast-induced hypersensitivity reactions, or any major concurrent neurological disorders (such as multiple sclerosis, intracranial tumors, or a history of stroke with persistent neurological deficits) were excluded, as these conditions could confound the interpretation of cerebral perfusion data. Additionally, individuals who were pregnant or breastfeeding were excluded due to the potential risks of contrast administration to the fetus or infant.

The study was conducted in accordance with the Declaration of Helsinki. This study received ethical approval from the Research Ethics Committee, Faculty of Allied Health Sciences, The University of Lahore, Punjab, Pakistan (Ref No: REC-UOL-680-06-2024), on 15 June 2024, for involving humans. Informed consent was obtained from all participants, and confidentiality was maintained regarding personal and medical information.

### 2.3. Outcome Variables

(a) Dependent Variables: The primary dependent variables in this study were CBF, measured in mL/100 g/min, and CBV, measured in mL/100 g. These perfusion parameters were quantified using DCE-MRI-derived perfusion maps.
(b) Independent Variables: The independent variables were the anatomically defined regions of interest (ROIs) corresponding to major cerebral arterial territories, including the MCA, ACA, and PCA, evaluated bilaterally. These ROIs were identified based on standard anatomical landmarks and applied consistently across all subjects.

### 2.4. Data Collection Tools and Procedures

Data collection was conducted using a (Toshiba Vantage Galan 3.0 Tesla MRI, Tochigi, Japan) equipped with a phased array head coil for high-resolution cerebral imaging. The imaging protocol included a pre-contrast T1-weighted sequence, a DCE T1-weighted sequence, and, optionally, a post-contrast T1-weighted sequence for anatomical referencing.

For the DCE-MRI sequence, imaging parameters were optimized to enhance the visualization of contrast perfusion across cerebral territories, with a flip angle of 30°, a repetition time (TR) of 3.5 ms, an echo time (TE) of 2.0 ms, and a field of view (FOV) of 220 mm. Additional acquisition parameters included a slice thickness of 5 mm, a matrix size of 256 × 256, and a dynamic series capturing approximately 60 to 80 time points over 4 to 5 min. The selected imaging parameters were optimized to maximize the resolution and sensitivity of the DCE-MRI sequence for assessing regional perfusion in ischemic stroke. A 5 mm slice thickness was chosen to balance spatial resolution with signal-to-noise ratio, allowing for precise visualization of perfusion deficits while minimizing scan time. A flip angle of 30° was selected to optimize tissue contrast without compromising signal intensity. The repetition time (TR) and echo time (TE) were chosen to optimize the contrast enhancement dynamics and temporal resolution for perfusion imaging. The chosen field of view (FOV) and matrix size ensure that all key cerebral regions are adequately covered while maintaining high-quality imaging for accurate perfusion measurements.

The DCE-MRI technique utilizes a T1-weighted sequence because it is highly sensitive to the T1-shortening effect of the gadolinium-based contrast agent. This paramagnetic agent reduces the T1 relaxation time of blood and perfused brain tissue, causing a transient increase in signal intensity on serial T1-weighted images. This temporal enhancement is directly proportional to the local concentration of the contrast agent and forms the basis for calculating perfusion parameters such as CBF and CBV.

The contrast agent used was Omnivist (Gadopentetate Dimeglumine), administered intravenously via the antecubital vein at a dose of 0.2 mL/kg body weight. The agent was delivered at a rate of 3 mL/s using a (MEDRAD Spectris Solaris EP power injector, Whippany, NJ, USA) followed by a saline flush.

The DCE-MRI T1-weighted images were first reconstructed on the scanner console to generate colour-coded perfusion maps representing CBF and CBV. The scanner’s built-in software produced these maps immediately after acquisition, and they were displayed on a red-to-blue scale indicating relative perfusion patterns.

The exported color-coded images were then post-processed from the acquired DCE-MRI T1-weighted images using dedicated perfusion analysis software (Olea Sphere 3.0, Olea Medical, La Ciotat, France). Motion correction was first applied to the dynamic image series to ensure alignment across all time points. To standardize the perfusion analysis, an arterial input was defined by placing a small region of interest in the M1 segment of the MCA, a location chosen for its reliable representation of arterial contrast arrival. Tissue regions of interest (ROIs) were then manually delineated by an experienced radiologist on co-registered T1-weighted images within the core vascular territories of the contralateral MCA, ACA, and PCA, guided by standard anatomical landmarks.

CBF and CBV quantitative values were extracted based on contrast enhancement dynamics and basic pharmacokinetic modelling principles. In this semi-quantitative approach, the signal enhancement over time caused by the passage of the gadolinium-based contrast agent was analyzed within defined regions of interest (ROIs) corresponding to the MCA, ACA, and PCA territories in both hemispheres. So, the CBV was determined from the time-integrated concentration of contrast agent within the tissue over the full acquisition period, normalized to the arterial input signal. CBF was derived from the maximum rate of contrast uptake in the tissue, also normalized to the arterial input. The final extracted values of CBF and CBV for each territory were further studied for statistical analysis.

### 2.5. Statistical Methods and Analysis

All statistical analyses were conducted using IBM SPSS Statistics for Windows, Version 29.0 (IBM Corp., Armonk, NY, USA). Descriptive statistics, including means, medians, standard deviations, and ranges, were calculated to summarize participants’ demographic characteristics and perfusion metrics (CBF and CBV).

To assess whether differences in CBF existed between contralateral arterial territories in the left and right hemispheres (i.e., right vs. left MCA, ACA, and PCA), paired-samples *t*-tests were conducted separately for each pair of arteries. This assesses hemispheric asymmetries via contralateral comparisons. A Bonferroni correction was applied to account for multiple comparisons, setting the adjusted significance threshold at (α = 0.05/3 = 0.0167).

To analyze whether CBF values varied between different arterial regions (MCA, ACA, and PCA) overall (irrespective of side), a one-way analysis of variance (ANOVA) was used. This allowed for the comparison of CBF across independent cerebral arterial territories (such as the MCA, ACA, and PCA) to detect inter-arterial CBF variations in the brain, irrespective of the hemisphere. The analysis focused on identifying an overall difference, and therefore, post hoc comparisons were not conducted.

To evaluate the relationship between CBF and CBV, both Pearson’s correlation coefficient (for normally distributed data) and Spearman’s rank correlation coefficient (for non-parametric data) were computed to assess how closely CBF and CBV are related. The *p*-values were evaluated against a Bonferroni-corrected threshold (α = 0.05/ 2 = 0.025) to account for the two correlation tests performed.

Finally, a multiple regression analysis was conducted to explore the combined effect of age and gender on CBF, with these demographic factors treated as independent variables and CBF as the dependent variable. This analysis enabled the evaluation of how variability in CBF could be attributed to age and gender differences but did not conduct a subgroup analysis comparing younger (e.g., 20–50 years) and older (e.g., 51–80 years) participants, prioritizing the continuous model’s robustness with our 55-patient sample. A two-tailed *p*-value < 0.05 was considered statistically significant for all tests, with Bonferroni-adjusted thresholds applied as specified above for the relevant multiple comparisons.

## 3. Results

### 3.1. Ischemic Stroke Patients’ Demographics and Clinical Presentation

A total of 55 patients diagnosed with acute ischemic stroke were included in this study. The demographic characteristics of the 55 participants are summarized in Table 1. The cohort had a broad age range (20–80 years; mean 52.0 ± 18.30 years) and comprised 35 males (63.6%) and 20 females (36.4%), with comparable age distributions between genders. This demographic variability allows for the assessment of age-related influences on cerebral perfusion parameters.

The variability in age allows us to assess how age influences CBF and CBV in ischemic stroke patients, providing insights into potential age-related perfusion changes and their impact on ischemic stroke patients.

Clinical symptomatology observed at the time of presentation is detailed in Table 2. The most frequent symptom was sudden unilateral weakness (18.2%), consistent with the characteristic motor deficits of ischemic stroke. Vestibular and coordination symptoms were equally prevalent (18.2%), followed by speech impairment (14.5%) and vision problems (12.7%).

These findings reflect the diverse neurological manifestations of ischemic stroke, which are crucial for understanding how symptomatology may reflect perfusion differences across cerebral arterial territories.

### 3.2. Cerebral Blood Flow (CBF) and Cerebral Blood Volume (CBV) Measurements

Descriptive statistics for CBF and CBV across the cohort of 55 ischemic stroke patients are provided in Table 3. The analysis revealed a moderate degree of variability in CBF values, a common finding in ischemic stroke, which may reflect differences in collateral circulation and the severity of ischemic injury. In contrast, CBV values showed relatively low dispersion around the mean.

The moderate degree of variability in CBF is common in ischemic stroke, as perfusion in certain regions may fluctuate due to factors such as collateral circulation, tissue viability, and the severity of ischemic injury. This variability in perfusion metrics is crucial for assessing stroke severity, as higher CBF and CBV values are typically associated with better tissue viability and less severe ischemic injury. Conversely, significantly lower CBF and CBV values could indicate areas of irreversible tissue damage, which have important implications for stroke prognosis and recovery.

These findings provide a quantitative baseline of central tendency and variability in cerebral perfusion among patients with ischemic stroke. The measured CBF and CBV values are within the expected physiological ranges for ischemic stroke patients, providing a baseline for subsequent comparative and correlation analyses across arterial territories and demographic subgroups.

### 3.3. Regional Cerebral Perfusion Patterns

**Figure 2:**
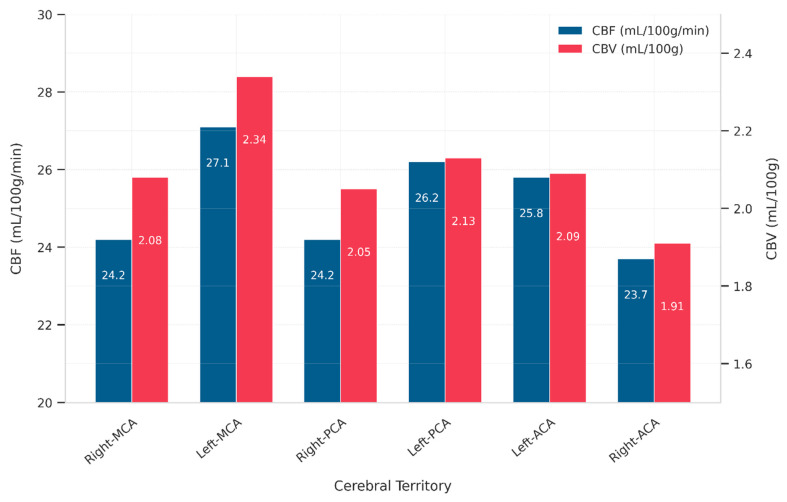
Mean cerebral blood flow (CBF) and cerebral blood volume (CBV) values across major cerebral arterial territories (MCA, ACA, PCA), illustrating both contralateral asymmetries between left and right hemispheres and inter-territorial variations irrespective of hemisphere. The graph visually confirms the left-sided perfusion dominance indicated by the data ranges in Table 4.

**Figure 3:**
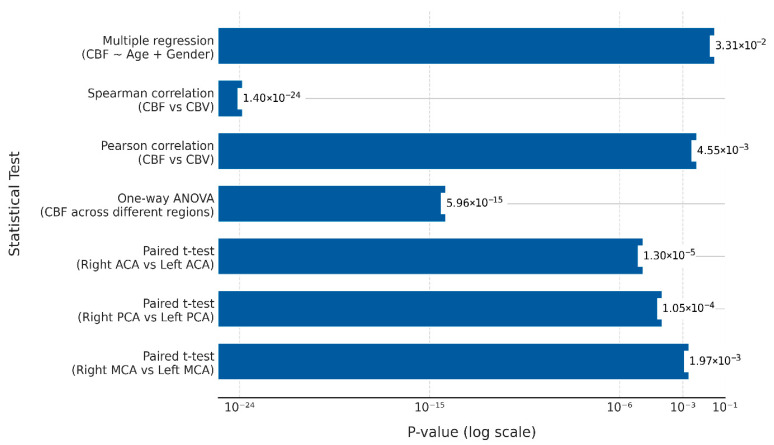
A horizontal bar graph displaying the *p*-values of statistical tests for cerebral perfusion differences, the relationship between cerebral blood flow (CBF) and cerebral blood volume (CBV), and the effects of age and gender on CBF in ischemic stroke patients. The *x*-axis represents *p*-values on a logarithmic scale. The *y*-axis lists the statistical tests conducted: paired *t*-tests used to assess whether significant differences in perfusion existed between contralateral arterial territories (Right Middle Cerebral Artery (MCA) vs. Left MCA, Right Posterior Cerebral Artery (PCA) vs. Left PCA, and Right Anterior Cerebral Artery (ACA) vs. Left ACA), one-way Analysis of Variance (ANOVA) for variations in CBF across different cerebral regions irrespective of the hemisphere, Pearson and Spearman correlation coefficients for assessing the relationship between CBF and CBV, and multiple regression analysis to evaluate the combined effect of age and gender on CBF. This figure illustrates the significance of perfusion differences between contralateral and different cerebral arterial regions, irrespective of hemisphere side, the strong association between CBF and CBV, and the impact of demographic factors on cerebral perfusion in ischemic stroke patients.

**Figure 4:**
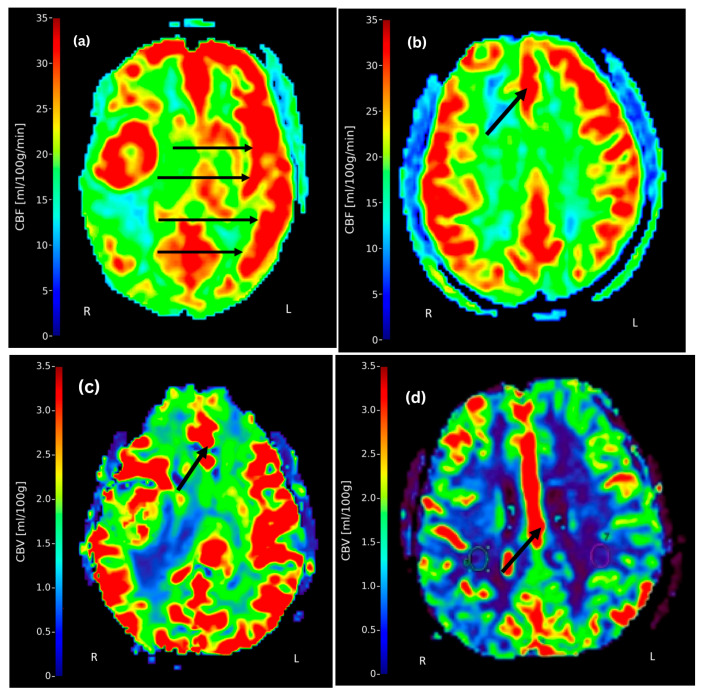
Representative post-contrast DCE-MRI T1-weighted axial slices acquired within 48 h of stroke symptom onset from four different ischemic stroke patients. Panels (**a**–**d**) display colour-coded perfusion maps quantifying cerebral blood flow (CBF; range: 0–35 mL/100 g/min) and cerebral blood volume (CBV; range: 0–3.5 mL/100 g). The parameters used for image acquisition and their post-processing are described in Section 2.4. (**a**) Image from a 63-year-old patient showing left-sided dominance of CBF in the middle cerebral artery (MCA) territory, as indicated by arrows. (**b**) Image from a 51-year-old patient showing elevated CBF in the left anterior cerebral artery (ACA) territory (arrow). (**c**,**d**) images from 26- and 75-year-old patients, respectively, showing high CBV in the left ACA territory (arrow). These visual findings are consistent with the results of the paired *t*-test analyses, which demonstrated significant hemispheric asymmetries in CBF and the descriptive analysis of CBF and CBV. The arrows highlight perfusion dominance or reduction regions, visually confirming the hemispheric asymmetries and perfusion variations quantified through descriptive (Table 3 and Table 4) and inferential (Table 5) statistical analyses.

The range of CBF and CBV across major cerebral arterial territories is presented in Table 4, with mean values visualized in Figure 2. The data demonstrate measurable perfusion variability between the left and right hemispheres, as well as across different vascular territories. A descriptive analysis of CBF and CBV values indicated a consistent pattern of left-sided dominance, which was particularly evident in the MCA and ACA regions as shown in Figure 3. Representative DCE-MRI perfusion maps illustrating these patterns are shown in Figure 4, where panels (a) and (b) highlight elevated CBF in the left MCA and ACA territories, respectively. Panels (c) and (d) demonstrate lateralization of CBV, with reduced perfusion in the right ACA territory, visually confirming the descriptive trends reported in Table 4. These asymmetrical perfusion patterns suggest hemodynamic differences between hemispheres that may correlate with stroke lateralization, which may vary with stroke location and severity, warranting further investigation to determine clinical significance.

### 3.4. Statistical Test Results: Perfusion and Demographic Relationships

The statistical analysis demonstrated significant findings related to contralateral hemispheric asymmetries, inter-territorial variations, the correlation between CBF and CBV, and the impact of demographic factors (age and gender), as summarized in Table 5.

Paired *t*-tests conducted between contralateral arterial regions (i.e., left and right MCA, ACA, and PCA) revealed statistically significant differences in CBF for all comparisons (right vs. left MCA: *p* = 0.00197; right vs. left PCA: *p* = 0.000105; right vs. left ACA: *p* = 0.000013). All *p*-values remained significant after applying a Bonferroni correction for multiple comparisons (adjusted α = 0.0167), confirming robust lateralization of CBF across hemispheres in ischemic stroke patients. These findings are visually supported by the perfusion maps in Figure 4, which demonstrate left-sided dominance in CBF. These results from the paired *t*-tests highlight the lateralization of CBF between contralateral hemispheres.

Separately, a one-way ANOVA was conducted to assess inter-territorial variation, testing for an overall difference in CBF values across the different cerebral arterial territories (MCA, ACA, PCA), irrespective of the hemisphere. The analysis indicated a significant overall difference. As the study’s objective was to identify the presence of this overall variability rather than to compare specific cerebral arterial territories against one another, post hoc comparisons (e.g., MCA vs. ACA) were not conducted.

A strong positive correlation was observed between CBF and CBV, as evidenced by both Pearson’s (r = 0.976) and Spearman’s (r = 0.928) correlation coefficients. Both correlations remained statistically significant after applying a Bonferroni correction for multiple comparisons (adjusted α = 0.025), with *p*-values for both tests being well below this threshold (*p* < 0.025), underscoring a robust interdependence between the two perfusion parameters. These findings suggest that as CBF increases, CBV also tends to increase.

The multiple regression analysis evaluating the combined effect of age and gender on CBF was statistically significant (R^2^ = 0.643), indicating that these demographic factors were significant predictors of cerebral perfusion in the studied cohort.

These results are consistent with the statistical findings presented in Figure 2, where the *p*-values highlight the significance of the observed relationships and differences in ischemic stroke patients.

## 4. Discussion

This study aimed to assess cerebral perfusion dynamics, specifically CBF and CBV, in ischemic stroke patients using DCE-MRI. Our findings revealed significant overall inter-territorial variations in CBF across major cerebral arteries (MCA, ACA, PCA) and descriptive variations in CBV across major cerebral arteries, as well as pronounced hemispheric asymmetries between the left and right hemispheres. These results confirm the presence of lateralized perfusion patterns in ischemic stroke, a phenomenon that may be vital for understanding stroke progression and therapeutic interventions. These results align with previous research, reinforcing the concept of lateralized perfusion patterns in ischemic stroke. Additionally, a strong positive correlation was identified between CBF and CBV, emphasizing the interdependence of these two perfusion parameters in stroke evaluation.

Age and gender both had significant effects on CBF in this study cohort. These findings offer valuable insights into the regional perfusion characteristics of ischemic stroke and highlight the potential of DCE-MRI as a reliable diagnostic tool, especially in settings with limited access to advanced imaging technologies.

### 4.1. Key Findings and Their Interpretation

This study revealed significant overall inter-territorial variation in CBF across the major cerebral arteries (MCA, ACA, PCA), as determined by a one-way ANOVA, irrespective of hemisphere. A descriptive analysis of CBV also indicated a consistent pattern of variation across these territories.

A separately paired *t*-test demonstrated contralateral hemispheric asymmetry with higher CBF values in the left hemisphere. The robustness of this asymmetry finding was confirmed, as all paired *t*-test comparisons remained significant after applying a Bonferroni correction (adjusted α = 0.0167).

These findings align with established research indicating lateralized perfusion abnormalities in ischemic stroke; however, such patterns may be influenced by unexamined factors, such as stroke location and severity, which were not evaluated in this study and limit inferences about causality or hemispheric implications.

The strong correlation between CBF and CBV (Pearson’s r = 0.976, Spearman’s r = 0.928) emphasizes the interdependence of these two parameters in assessing stroke severity. Notably, this strong relationship was maintained after applying a Bonferroni correction for the two correlation tests performed (adjusted α = 0.025). CBF and CBV provide complementary information about tissue viability and the extent of ischemic damage, reinforcing their importance in stroke assessment. This relationship reflects the dynamic coupling of blood flow and volume during ischemic events, a phenomenon that is central to understanding stroke pathophysiology. Clinically, this coupling can inform early assessments of tissue viability, enabling clinicians to predict stroke severity and formulate effective treatment strategies.

Regarding age, our findings revealed that age significantly contributes to variability in CBF. While some studies suggest a linear decrease in CBF with increasing age, our results indicate a more complex relationship between age and cerebral perfusion. This study did not examine the evaluation of age effects across categorized groups; however, further research into this area is warranted. Future studies could address this gap, potentially informing customized treatment strategies for elderly stroke patients and enhancing individualized care in clinical practice.

Interestingly, our study observed that gender also affected CBF, which contrasts with some studies suggesting minimal gender-based vascular differences. This finding highlights the importance of considering gender as a factor in cerebral perfusion dynamics, particularly in ischemic stroke. Discrepancies between studies may arise from variations in patient cohorts, methodology, or the specific focus of the studies. Future research with larger, more diverse populations will be essential to better understand the role of gender in cerebral perfusion and stroke pathophysiology.

### 4.2. Comparison with Existing Literature

#### 4.2.1. Use of DCE-MRI for Perfusion Imaging

Our study, as well as that of Sasannia et al. [12], demonstrated the clinical utility of DCE-MRI for assessing CBV and CBF in ischemic stroke. Sasannia et al. [12] highlighted that DCE-MRI can detect early perfusion changes, even before conventional imaging modalities, such as DWI and ASL, become apparent. Our study aligns with these findings, reinforcing the growing body of evidence that DCE-MRI is a highly sensitive and reliable modality for evaluating stroke severity and ischemic tissue viability. This early detection capability is critical in guiding timely interventions and improving patient outcomes.

In addition, the study by Lu et al. [22], explored the application of both DCE-MRI and Dynamic Susceptibility Contrast MRI (DSC-MRI) for evaluating vascular and hemodynamic features in acute ischemic stroke. Their findings align with our research, underscoring the importance of advanced imaging techniques in assessing stroke severity and informing clinical decision-making. Lu et al. [22] also highlighted the superiority of DCE-MRI in detecting subtle perfusion abnormalities, a key observation that resonates with our identification of regional perfusion deficits in ischemic stroke. Both studies emphasize the clinical utility of DCE-MRI in elucidating the complex pathophysiology of stroke, as this imaging modality offers valuable insights into the brain’s vascular dynamics and facilitates the identification of at-risk brain regions.

#### 4.2.2. Inter-Territorial and Hemispheric Perfusion Variations

Our findings align with those of Valente et al. [26], who observed regional perfusion abnormalities across the MCA, ACA, and PCA territories in ischemic stroke patients. Using CT vascular territory mapping, Valente et al. [26] identified collateral circulation in patients with large vessel occlusion (LVO), finding significant perfusion deficits in the MCA, ACA, and PCA regions. This is consistent with our study’s observation of asymmetries in CBF and CBV between hemispheres, particularly between the left and right hemispheres. These findings underscore the clinical relevance of regional perfusion differences in stroke assessment, highlighting the value of detailed perfusion imaging in understanding the pathophysiology of stroke.

Furthermore, Furlanis et al. [27] reinforce the utility of advanced perfusion imaging, particularly CT perfusion (CTP), in detecting acute posterior circulation stroke (PCS) and identifying associated clinical factors. Their work contributes to the growing body of evidence supporting the clinical importance of perfusion metrics in assessing ischemic stroke, emphasizing the need for personalized treatment strategies based on perfusion abnormalities. Our research echoes these findings, further validating the role of advanced imaging in stroke management.

The study by Strinitz et al. [28] provides additional support for the clinical significance of CBV, showing that higher relative cerebral blood volume (rCBV) in the affected brain region is associated with better long-term functional outcomes in acute ischemic stroke. Their retrospective cohort study demonstrated that elevated rCBV, even after adjusting for baseline stroke severity and collateral circulation, correlates with improved prognosis. This aligns with our observation that regional variations in CBV and CBF are strongly linked to functional outcomes, reinforcing the importance of these biomarkers in determining stroke severity and guiding treatment decisions.

#### 4.2.3. Perfusion Metrics and Their Relationship to Brain Volume

In line with Kikuchi et al. [29], our study suggests that regional perfusion variations are key indicators of brain tissue viability and stroke severity. Kikuchi et al. [29] reported that perfusion measurements correlate strongly with brain volume, particularly in areas such as the frontal and temporal lobes, which are crucial for cognitive function. Our findings further support this, as variations in CBV and CBF in specific brain regions were associated with functional outcomes, underscoring the importance of perfusion metrics in assessing the health of brain tissue post-stroke.

#### 4.2.4. Age and Gender Effects on CBF in Ischemic Stroke

This study revealed that both age and gender significantly influence CBF in ischemic stroke patients, although the specific patterns or age groups most affected were not explored. Our findings suggest that age contributes to variations in CBF, supporting the notion that age-related vascular changes may be a key factor influencing perfusion in ischemic stroke. This is consistent with previous studies by Shao et al. [30] and Hu et al. [31], which highlighted age-related perfusion changes. However, the exact age group most affected by these changes remains complex, as our study observed a nuanced relationship between age and CBF, rather than a simple linear decrease.

In terms of gender, our study found that gender is a factor affecting CBF, which contrasts with some studies, such as Shao et al. [30], which suggest that males typically exhibit lower CBF values, particularly due to age-related vascular decline. The lack of significant findings in some studies could be due to methodological differences, sample sizes, or the focus on ischemic stroke in our cohort. Studies with broader stroke populations, like Shao et al. [30], may have observed different gender-related effects on CBF, whereas our research concentrated specifically on ischemic stroke patients.

### 4.3. Implications for Future Research

Future research should address the limitations of this study, such as the small sample size, exclusion of certain groups, and the omission of potential confounders (e.g., stroke volume, lesion location, and time since onset) in the multiple regression analysis. These gaps may have influenced the multiple regression analysis, necessitating the use of larger, more diverse cohorts across multiple centers to validate findings and improve generalizability. In such larger-scale studies, the reporting of effect sizes and confidence intervals will be crucial to provide a more nuanced understanding of the magnitude and precision of the observed perfusion differences. Additionally, the effects of age, categorized into groups, were not assessed, as our approach utilized a continuous age variable. Investigating underlying comorbidities and their influence on cerebral perfusion, alongside these confounders, could refine future regression models, providing deeper insights into stroke pathophysiology and supporting tailored treatment strategies for diverse patient profiles.

## 5. Conclusions

In this study, we assessed cerebral perfusion dynamics in ischemic stroke patients using DCE-MRI, revealing inter-territorial variations in CBF and CBV across cerebral arterial territories (regardless of hemisphere), along with notable hemispheric asymmetries that showed left-sided dominance. This finding supports established research demonstrating lateralized perfusion patterns in ischemic stroke. Additionally, a strong positive correlation was observed between CBF and CBV, highlighting their interdependence as essential parameters for assessing stroke severity and tissue viability.

According to the multiple regression analysis, both age and gender were found to influence CBF in ischemic stroke patients significantly. However, the specific patterns of how these factors affect CBF were not explored in detail. These results underscore the importance of considering age and gender when assessing cerebral perfusion, paving the way for future research that could provide a more nuanced understanding of their effects and ultimately inform personalized stroke management strategies.

The clinical implications of this study are significant. Our results underscore the potential of DCE-MRI as a reliable diagnostic tool for assessing regional perfusion, especially in settings with limited access to advanced imaging technologies. By addressing gaps in regional and demographic perfusion analysis, our study provides valuable insights into stroke imaging, offering directions for more targeted and personalized treatment strategies.

However, the study has limitations, including its sample size and the exclusion of specific patient groups, which may affect the generalizability of the results. A limitation of this study is the absence of analysis on how stroke location and severity influence hemispheric asymmetries (from contralateral comparisons) and inter-territorial variations, which may impact the interpretation of perfusion patterns. Furthermore, the multiple regression analysis of demographic influences (age and gender) was preliminary and did not include clinical confounders and the effect of age categorized into groups, limiting the generalizability of the findings. Future research with larger, more diverse cohorts and comprehensive clinical data is essential to validate these perfusion dynamics and should incorporate advanced statistical measures, such as effect sizes and confidence intervals, to build a more robust and generalizable statistical analysis. Such efforts will be crucial for refining stroke management and advancing toward truly personalized treatment interventions for ischemic stroke patients.

## Figures and Tables

**Table 1:** Demographic Summary and Age Distribution of 55 Ischemic Stroke Patients.

Demographic Characteristic	Overall	Male	Female
**Total Number of Patients n (%)**	55 (100%)	35 (63.6%)	20 (36.4%)
**Age Range (Years)**	20–80	20–80	22–77
**Mean Age ± SD (Years)**	52.0 ± 18.30	51.6 ± 18.08	52.8 ± 17.40
**Median Age (Years)**	55.0	56.0	55.5

**Table 2:** Frequency of Clinical Symptoms in 55 Ischemic Stroke Patients.

Symptom	n (%)
Sudden Weakness on One Side	10 (18.2)
Vestibular & Coordination Symptoms	10 (18.2)
Speech Impairment	8 (14.5)
Vision Problems	7 (12.7)
Confusion	6 (10.9)
Facial Drooping	5 (9.1)
Nausea	5 (9.1)
Severe Headache	4 (7.3)

**Table 3:** Descriptive Statistics for Cerebral Blood Flow (CBF) and Cerebral Blood Volume (CBV) in Ischemic Stroke Patients.

Parameters	Mean	Median	Minimum	Maximum	Standard Deviation (±SD)
**CBF (mL/100 g/min)**	25.39	25	20	32	±3.15
**CBV (mL/100 g)**	2.08	2.0	1.6	2.7	±0.29

CBF: Cerebral Blood Flow (CBF); CBV: Cerebral Blood Volume (CBV); SD: Standard Deviation (SD).

**Table 4:** Range of Cerebral Blood Flow (CBF) and Cerebral Blood Volume (CBV) Across Different Cerebral Arterial Territories in Ischemic Stroke Patients.

Parameter	Right-MCA Territory	Left-MCA Territory	Right-PCA Territory	Left-PCA Territory	Right-ACA Territory	Left-ACA Territory
**CBF (mL/100 g/min)**	Range: 20–29	Range: 22–32	Range: 21–30	Range: 24–28	Range: 20–27	Range: 23–29
**CBV (mL/100 g)**	Range: 1.8–2.4	Range: 2.0–2.7	Range: 1.7–2.5	Range: 2.0–2.4	Range: 1.7–2.1	Range: 1.9–2.4

CBF: Cerebral Blood Flow; CBV: Cerebral Blood Volume; MCA: Middle Cerebral Artery; PCA: Posterior Cerebral Artery; ACA: Anterior Cerebral Artery.

**Table 5:** Statistical Test Results for Cerebral Perfusion and Demographic Factors in Ischemic Stroke Patients.

Statistical Test	Statistic	*p*-Value
**Paired *t*-test (Right MCA vs** **. Left MCA)**	T-statistic: (−7.204)	0.00197
**Paired *t*-test (Right PCA vs** **. Left PCA)**	T-statistic: (−15.339)	0.000105
**Paired *t*-test (Right ACA vs** **. Left ACA)**	T-statistic: (−25.820)	0.000013
**One-way ANOVA (Cerebral regions)**	F-statistic: 93.847	5.96 × 10^−15^
**Pearson-Correlation (CBF vs. CBV)**	R: 0.976	0.00455
**Spearman-Correlation (CBF vs. CBV)**	R: 0.928	1.40 × 10^−24^
**Multiple Regression Analysis (CBF Age + Gender)**	R^2^: 0.643	F-stat: 0.0331

MCA: Middle Cerebral Artery (MCA); PCA: Posterior Cerebral Artery (PCA); ACA: Anterior Cerebral Artery (ACA); ANOVA: Analysis of Variance (ANOVA); CBF: Cerebral Blood Flow (CBF); CBV: Cerebral Blood Volume (CBV); R: Correlation Coefficient (R); R^2^: Coefficient of Determination (R^2^); F-stat: F-statistic (F-stat).

## Data Availability

All data supporting the findings of this study are included within the article.

## Abbreviations

The following abbreviations are used in this manuscript:

CBF	Cerebral Blood Flow
CBV	Cerebral Blood Volume
DCE-MRI	Dynamic Contrast-Enhanced Magnetic Resonance Imaging
MCA	Middle Cerebral Artery
ACA	Anterior Cerebral Artery
PCA	Posterior Cerebral Artery
CTP	Computed Tomography Perfusion
DSC-MRI	Dynamic Susceptibility Contrast Magnetic Resonance Imaging
LMICs	Low- and Middle-Income Countries
ANOVA	Analysis of Variance
SD	Standard Deviation
eGFR	Estimated Glomerular Filtration Rate
ROIs	Regions of Interest
